# Modification of the Raman Spectra in Graphene-Based Nanofluids and Its Correlation with Thermal Properties

**DOI:** 10.3390/nano9050804

**Published:** 2019-05-26

**Authors:** María del Rocío Rodríguez-Laguna, Pedro Gómez-Romero, Clivia M. Sotomayor Torres, Emigdio Chavez-Angel

**Affiliations:** 1 Catalan Institute of Nanoscience and Nanotechnology (ICN2), CSIC and The Barcelona Institute of Science and Technology (BIST), Campus UAB, Bellaterra, 08193 Barcelona, Spain; clivia.sotomayor@icn2.cat; 2 Department of Chemistry, Universitat Autònoma de Barcelona, Campus UAB, Bellaterra, 08193 Barcelona, Spain; 3 ICREA- Institució Catalana de Recerca i Estudis Avançats, Pg. Lluís Companys 23, 08010 Barcelona, Spain

**Keywords:** raman of nanofluids, enhancement of thermal conductivity, nanofluids, graphene

## Abstract

It is well known that by dispersing nanoparticles in a fluid, the thermal conductivity of the resulting nanofluid tends to increase with the concentration of nanoparticles. However, it is not clear what the mechanism behind this phenomenon is. Raman spectroscopy is a characterization technique connecting the molecular and macroscopic world, and therefore, it can unravel the puzzling effect exerted by the nanomaterial on the fluid. In this work, we report on a comparative study on the thermal conductivity, vibrational spectra and viscosity of graphene nanofluids based on three different amides: *N*, *N*-dimethylacetamide (DMAc); *N*, *N*-dimethylformamide (DMF); and *N*-methyl-2-pyrrolidinone (NMP). A set of concentrations of highly stable surfactant-free graphene nanofluids developed in-house was prepared and characterized. A correlation between the modification of the vibrational spectra of the fluids and an increase in their thermal conductivity in the presence of graphene was confirmed. Furthermore, an explanation of the non-modification of the thermal conductivity in graphene-NMP nanofluids is given based on its structure and a peculiar arrangement of the fluid.

## 1. Introduction

The continuous trend towards miniaturization in the electronics industry has led to smaller devices with more processing power. However, as dimension decreases, the effective thermal conductivity (*k*) of each component also decreases and, consequently, energy losses increase [1]. In the case of the emerging charge-based nanoelectronic switches, the density of these devices is limited by a maximum allowable power dissipation of ~100 W/cm^2^, and not by their size [2]. 

Therefore, it is a matter of high priority to design effective and novel cooling strategies, at a reasonable price to meet the cooling needs of the electronics industry. To guarantee proper performance and reliability of electronic components, excess heat must be carried away from critical components and hot spots, and then dissipated to the environment. Conduction and convection are the primary modes of heat transfer in cooling strategies of electronics, where radiation expels only a small part of the total thermal load. Conventional cooling strategies can be divided into active and passive. Passive cooling does not consume energy and is mainly based on natural convection, radiation, and heat conduction by utilizing heat spreader or a heat sink with very high thermal conductivity. Metal materials, such as aluminum, copper, zinc, are the most used as heat sinks [3]. Passive cooling technology relies on maximizing heat transfer by conduction, radiation, and convection using heat sinks, heat spreaders, heat pipes, or thermal interface materials to maintain optimal operating temperatures. These components can be found in all electronic devices and only require an architectural design of heat sinks and choice of the right material [4]. Active cooling, on the other hand, requires an external device (e.g., a fan) to force the cooling of the system. However, a major drawback of this technology is related to the consumption of extra energy to keep the device in operation although the rate of heat removal is much larger than that provided by passive cooling methods (except strategies using latent heat) [5]. Thus, active cooling can deal with higher power densities, and current cooling strategies include: Forced-air and forced-liquid cooling, direct liquid immersion, thermoelectric coolers, and forced convection [5,6,7].

The vast majority of active cooling systems include a heat sink or heat spreader in direct contact with the heat source. Excess heat is transported away from the electronic component by (1) conduction towards the cold edges of the solid piece or to a second piece (heat sink, pipe or cold plate) and then, by (2) forced convection in air or liquid. It is clear that the material, together with the structural and architectural design of heat sink, is crucial to increase efficiency levels of active cooling systems. Great efforts have been made to improve the performance of current heat sinks, such as the modification of the architectural designs [8,9,10,11], development of composite materials with enhanced thermal properties (metal-SiC, metal-graphene/diamond) [3,12,13,14], and study and control of the structure to enhance their thermal conductivity (e.g., porosity) [14]. Therefore, to improve the efficiency of active cooling systems, there must be an optimization of solid materials acting as heat sinks/spreaders/pipes and active components, such as fluids and those related to mechanical forces (fan, pump, etc.).

Among these active methods, the air-based (heat-sink-and-fan) can be found in many electronic devices from the cheapest laptop to the most expensive laboratory equipment. However, this is an old technology and not always efficient. Furthermore, it is not cost-effective for the dissipation of large amounts of heat. Murshed and Nieto de Castro [5] demonstrated that forced convection of air has very low heat removal rates compared with forced convection of liquids and that natural convection has the lowest heat removal rate among all cooling modes.

In this context, liquid-cooling technology has proven to be an efficient and low-cost method to cool high-power components [5,15,16], despite the potential risk of having liquid close to an electronic device. This becomes evident in the designs of high-density boards, where air cooling can become so challenging that the only viable option is the use of liquid cold plate technologies. [17] In any case, it is important to remark that the implementation of an effective cooling strategy in both high-power and low-power components may require the use of a combination of liquid- and air-cooling technologies, as well as a proper design of the passive components (heat sinks and heat spreaders).

Within liquid-based active cooling, the most common is the use of heat transfer fluids (or coolants) confined in basins. The coolant flows from tower pumps through pipes along the hot side of the device, thereby reducing the temperature.

Conventional coolants are mainly classified into dielectric and non-dielectric fluids. Dielectric coolants are organic-based fluids with quite low thermal conductivity but widely used in direct liquid immersion systems, while, the non-dielectric coolants are preferred for heat pipes and microfluidics due to their superior thermal properties compared to their dielectric counterparts. They are normally aqueous solutions and thus exhibit quite a high heat capacity and low viscosity. Water, ethylene glycol, and mixtures of these two are the most popular and widely used as coolants for many electronic devices [5]. However, a major problem of conventional coolants is the low heat exchange rate and thermal conductivity, which is too small to meet the coming needs and challenges in the field. One way to overcome this barrier is by using solid particles dispersed in fluids to improve their thermal properties [18,19,20].

Nanofluids (NFs) are colloidal dispersions of nanoparticles (1–100 nm) suspended in a base liquid [21]. The presence of well-dispersed nanoparticles in a fluid can provide remarkable improvements in thermal properties, such as thermal conductivity [18,22,23,24,25,26,27] and in some cases, specific heat capacity [27,28,29,30,31,32,33,34]. The heat transfer performance of a number of nanofluids has been tested compared to that of their base fluids in commercial electronic cooling units for CPUs and chips, as well as in heat pipes (passive cooling). The results are promising, showing an effective decrease in the temperature of the electronic components (CPUs and chips) [35,36,37,38,39] as well as an enhancement in the heat transfer performance of heat pipes [40,41,42,43,44].

Several studies have been reported on the enhancement of thermophysical properties in nanofluids, however, the mechanism behind this phenomenon is not clear [45,46,47,48,49]. In the literature, at least six different mechanisms have been suggested for explaining this enhancement: (*i*) Increase of the thermal transfer due to Brownian motion of nanoparticles, (*ii*) localized convection created in the fluid due to Brownian motion of nanoparticles, (*iii*) agglomeration of nanoparticles, (*iv*) enhanced thermal energy transfer due to increased interatomic interactions arising from the modification of interatomic potential, (*v*) ordered layering of liquid around the solid, and (*vi*) ballistic phonon transport of heat through solid nanoparticles [45,46,47,48,49]. In a previous report [27], we showed that for these kinds of fluids (amides) the mechanisms: (*i*), (*ii*) and (*iii*) cannot explain our experimental observations. In addition, the displacement of some Raman modes of DMF-graphene NFs led us to conclude that there is a strong modification of the interatomic potential as a function of graphene concentration, as the model (*iv*) suggests.

Taking into account all of the above, in this paper, we report an extension of our previous work related to the enhancement of thermophysical properties of graphene-based nanofluids [27]. Here, we present a thorough study of the vibrational properties of two previously reported NFs based on: N, N-dimethylacetamide (DMAc) and N, N-dimethylformamide (DMF) and we extend the work to a NF based on N-methyl-2-pyrrolidinone (NMP). The thermophysical properties of each NF are discussed based on the Raman observations.

## 2. Materials and Methods 

Graphene flakes were prepared from graphite (Sigma-Aldrich, purity > 99% and size < 20 um, St. Louis, MO, USA) by mechanical exfoliation. An extended description of the sample preparation can be found in the section on experimental methods in the supporting information of Reference [27]. The produced graphene size was around 150–450 nm and the number or layers varied from 2–10. *N*, *N*-dimethylacetamide (ACROS Organics, 99+%, Fair Lawn, NJ, USA), *N*, *N*-dimethylformamide (Scharlau, HPLC grade, Barcelona, Spain) and *N*-methyl-2-pyrrolidinone (Sigma Aldrich, 99.5%, St. Louis, MO, USA) were used as the base fluids. The preparation of the nanofluids consisted of direct mixing of the base fluid with graphene nanosheets. Then, graphene was dispersed in the fluid using a planetary ball mill (All-direction planetary ball mill 0.4 L, model CIT-XBM4X-V 0.4 L, Columbia International, Irmo, SC, USA) and soft ultrasonic vibration (Ovan, model ATM40-6LCD, Barcelona, Spain) for 1 hour. These techniques were used to suppress the formation of particle clusters in order to obtain stable dispersions [50]. The samples were later centrifuged for one hour at 6000 rpm to ensure the stability of the NFs. The stability of the dispersions was evaluated by periodic analyses using dynamic light scattering (DLS) as is described in [27].

The measurements of thermal conductivity of the nanofluids were carried out by using the well-known three-omega (3ω) method [51,52] in the bidirectional configuration [53,54]. An extended description of the setup can be found in the section on experimental methods in the supporting information of References [27,55]. The effective viscosity of the nanofluids was measured using a Haake RheoStress RS600 rheometer from Thermo Electron Corp. at *T* = 20–21 °C. The shear rate used was 2880 s^−1^ with a measurement time of 30 seconds. The Raman spectra were recorded by T64000 Raman spectrometer manufactured by HORIBA Jobin Yvon (Chilly-Mazarin, France). It was used in single grating mode (2400 lines) with a spectral resolution better than 0.4 cm^−1^. The NF was placed in a transparent quartz cuvette. Then a green diode laser (λ = 532 nm) was focused on the cuvette by using a 50× long working distance microscope objective. The power of the laser was kept as low as possible (<2 mW) to avoid any possible effect from self-heating. Raman analyses of the samples were repeated under similar conditions as a function of time (day 1, after ~one month and more than one year later) to verify the reproducibility of the Raman spectra (See Figures S1–S3 in the supporting information for more details).

## 3. Results and Discussions

### 3.1. Thermal Conductivity and Viscosity Measurements

The Table 1 shows the thermal conductivity for different graphene concentrations (C) in nanofluids based on *N*, *N*-dimethylformamide (DMF), *N*, *N*-dimethylacetamide (DMAc) and *N*-methyl-2-pyrrolidinone (NMP). Progressive enhancement of *k* was observed in DMAc and DMF-based nanofluids (NFs) as C increased, with a maximum enhancement of ~48% for *C* = 1.50 mg/mL in DMAc-based NF. Concentrations between 0.1–0.5 mg/mL in DMF and DMAc produced relatively large enhancements of ~6–25% and 3–17%, respectively. While in the same set of concentrations no significant enhancement of *k* was detected in NMP-based NFs. In contrast, in the case of viscosity, a maximum enhancement of ~44% was observed for 0.50 mg/mL graphene in NMP.

An increase in viscosity as the nanoparticle concentrations rise has been frequently observed in graphene-based [23,26,56,57,58,59] and other NFs [23,26]. This enhancement has been commonly associated with the tendency of the nanoparticles to agglomerate under pressure. The agglomerates raise the internal shear stress and the resistance to flow in the NF, which leads to an increase in viscosity during measurement [58].

### 3.2. Raman Measurements

Firstly, we will discuss the Raman characterization of DMAc and DMF as a function of graphene concentration ranging from 0 < C < 1.5 and 0 < C < 1.13 mg/mL, respectively. Due to the very high light absorption of the most concentrated dispersions (C = 1.5 and 1.13 mg/mL for DMAc and DMF, respectively), we were only able to detect one peak in the entire Raman spectrum. The vibrational assignment for DMAc and DMF was done based on the work of Chalapathi and Ramiah [60]. Secondly, we will show and discuss the Raman spectra of NMP-NFs for three different graphene concentrations of *C* = 0, 0.05 and 0.10 mg/mL. The vibrational assignment was carried out based on the work of Peek and McDermott [61] and Xu et al. [62]. The peak positions of all the NFs were estimated using a Lorentzian fit considering a constant background. Raman analyses of the samples were repeated under similar conditions as a function of time: Day 1, after ~one month and one year later. We found excellent reproducibility of the Raman spectra of DMAc, DMF, and NMP-nanofluids as can be seen in Figures S1–S3 that are shown in the supporting information. These results confirm the high stability of these dispersions over time and are in agreement with previously performed DLS analyses [27].

#### 3.2.1. Raman of DMAc- and DMF-Based Nanofluids

##### Graphene-DMAc Nanofluids

**Figure 1:**
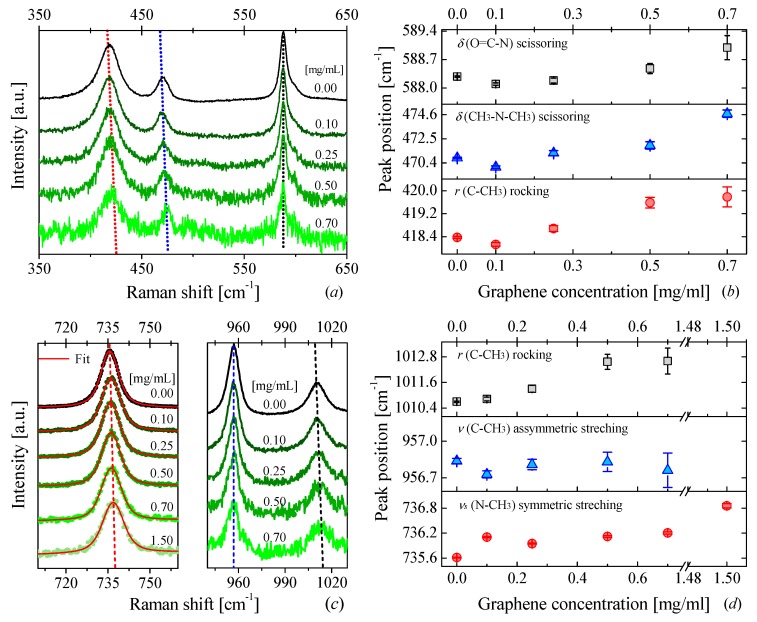
Raman scattering of DMAc-based NFs and peak positions of several Raman bands: (**a**,**c**) Raman spectra of DMAc-based NFs for different graphene concentrations, (**b**,**d**) peak positions of six Raman bands shown in (**a**,**c**) as a function of graphene concentration.

The Raman spectra of DMAc for different graphene concentrations are displayed in Figure 1a,c. A clear blue-shift and a broadening of the Raman bands can be observed as the graphene concentration increases. The largest displacement was found to be ~4 cm^−1^ for a band around ~470 cm^−1^ (see Figure 1b). This band corresponds to a scissoring vibration from the bond (CH_3_–N–CH_3_) of the DMAc molecule [60]. The displacement of this and the other bands to higher frequencies in the presence of graphene is an indicator of a strong modification of interatomic forces within the nanofluid.

##### Graphene-DMF Nanofluids

**Figure 2:**
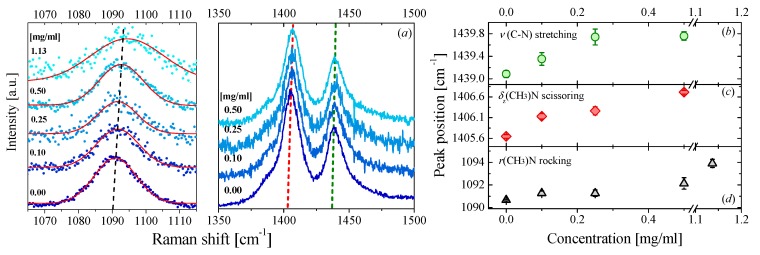
Raman scattering of DMF-based NFs and peak positions of several Raman bands: (**a**) Raman spectra of DMF-based NFs for different graphene concentrations, (**b**–**d**) peak positions of three Raman bands shown in (**a**) as a function of graphene concentration.

A similar effect can also be observed in DMF-based nanofluids as displayed in Figure 2b,d Here, the largest displacement ~4 cm^−1^ occurred for a rocking vibration of the bond (CH_3_)–N of the DMF molecule. In a previous study, we related such observation to an apparent hardening of the bonds of DMF molecules produced by the presence of the graphene flakes. [27] In this study, simulations based on density functional theory (DFT) and molecular dynamics (MD) suggested that the presence of the graphene flake acts as an external force field that induces a parallel orientation of the DMF molecules in the proximity of the graphene flakes.

Taking into account that both graphene and DMF molecules contain a *p*-orbital with unshared electron pairs (delocalized electrons) along the out-of-plane direction [63,64]. The parallel orientation of the DMF molecules with respect to the graphene flakes can favor the overlap of two adjacent *p*-orbitals. It can favor a *π*-*π* bond between the solvent molecules and the graphene surface. This interaction thus increases the rigidity of the internal bonds of the molecules closest to graphene and, consequently, modifies their Raman spectra. It is important to mention that our simulations suggested that this interaction is local rather than global, i.e., just the molecules around of graphene flakes feel this change. It is not clear how the rest of the fluid is also affected by this interaction. Returning to DMAc NFs, a similar blue-shift in several Raman modes can be observed. If we consider that DMF and DMAc molecules are amides with quite similar structures and electron delocalization, then it is reasonable to suggest that a similar geometric configuration occurs in DMAc-based NFs. As a consequence, DMAc molecules may also tend to lay parallel to the graphene flakes, favoring a *π*-*π* stacking between the graphene and the molecules thereby contributing to the blue-shift in the Raman spectra.

#### 3.2.2. Raman in NMP-Based Nanofluids

**Figure 3:**
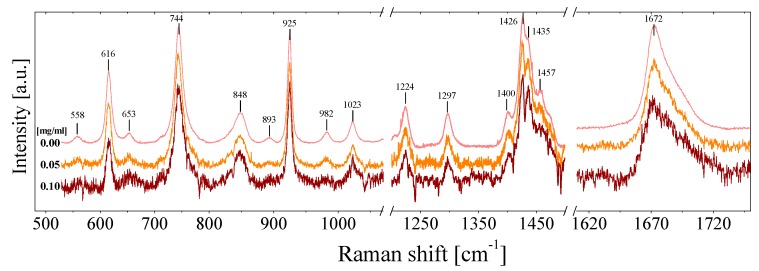
Raman spectra of NMP-based nanofluids for three different graphene concentrations.

**Figure 4:**
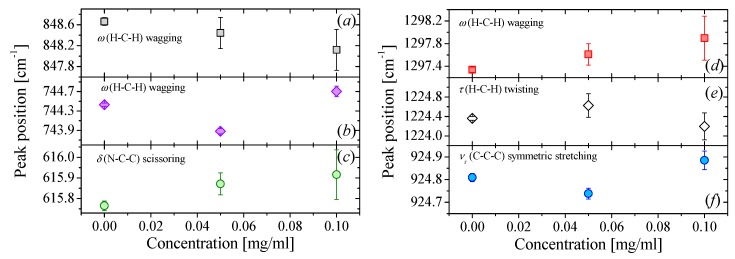
(**a**–**f**) Peak positions of six Raman bands of NMP shown in Figure 3 as a function of graphene concentration.

The case of NMP-based fluids is quite different, Figure 3 shows the full Raman spectra of NMP-based NFs for three different graphene concentration of *C* = 0, 0.05 and 0.10 mg/mL. At first glance, a very small displacement of certain Raman modes can be detected (see Figure 4). However, the displacement is both within the range of the spectral reproducibility of the equipment (~0.25 cm^−1^) and the error determination of each peak position. In conclusion, there is not a significant displacement of the Raman bands as the graphene concentration increases.

The Raman spectroscopy results from graphene-NMP NFs are not surprising if we consider the structure of the organic compound. *N*-methyl-2-pyrrolidone is a five-membered cyclic amide (with a ring structure), and therefore, the bonds tend to be more rigid than those of amides having a linear structure. Consequently, it is conceivable that small amounts of graphene do not affect or modify the intramolecular vibrations of inner bonds in the solvent. Furthermore, Adams et al. [65] suggested that NMP molecules tend to spontaneously form dimers in the liquid state. NMP molecules strongly interact in pairs. Therefore, the impact of the presence of graphene flakes on the vibration of the bonds should be negligible. The findings of Adam et al. reinforce the idea that the solvent creates a natural barrier that prevents graphene from modifying the intramolecular interactions of the solvent. Moreover, Basma et al. [66] reported that pure NMP has a well-developed intrinsic order compared to other solvents. This inner order, together with the sp^2^ hybridization of N-C-O (which allows a possible *π*-*π* interaction between NMP and graphene), can explain the long-term dispersion stability of graphene in NMP. However, the internal structure of NMP seems to be an obstacle to the improvement of the thermal properties of the fluid induced, in other cases, by graphene.

**Figure 5:**
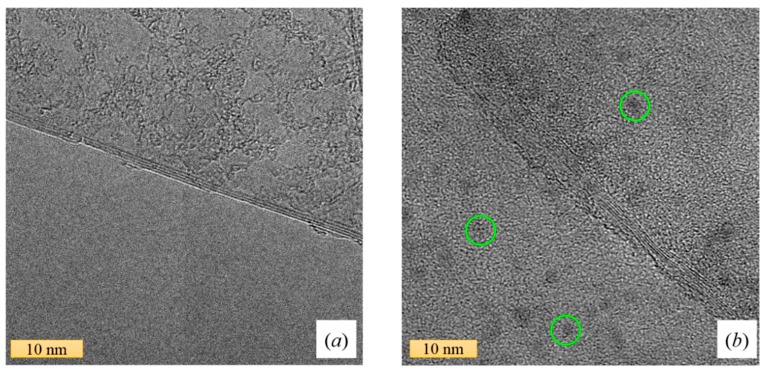
Representative transmission electron microscopy (TEM) images of graphene from: (**a**) a DMAc dispersion and (**b**) an NMP dispersion. Both images show the multilayer nature of the flakes folded graphene sheets. Highlighted by green circles, some nanoparticulates can be observed in the NMP-based nanofluid.

At this stage, one could consider as a possibility that higher concentrations of graphene may be preferable in order to observe a modification of the Raman spectra of NMP. However, although we prepared higher concentrations of this nanofluid, it was not possible to measure their Raman spectra. In addition to the high light absorption in the most concentrated samples, we also observed photoluminescence, which masks the Raman peaks. The origin of this photoluminescence has been attributed to the sonochemical degradation of the NMP suffered during the preparation of the NFs. Previous investigations [67,68] have demonstrated that sonication of pure NMP produces contaminating organic nanoparticles. Ogilvie et al. [68] also reported strong photoluminescence and enhancement of the optical absorbance of NMP-based NFs after a sonication treatment. In Figure 5a,b, we can see the transmission electron microscopy (TEM) images of two graphene flakes sonicated in DMAc and NMP, respectively. It is interesting to observe in Figure 5b small particles on the graphene flakes. The sample of the image corresponds to a nanofluid of graphene-NMP, which was sonicated during the preparation stage. Similar particles were also found by Yau et al. [67] after the sonication of dispersion of NMP and single-walled carbon nanotubes. Their experimental data suggested that the sonication leads to the polymerization of the fluid, producing contamination by small organic nanoparticulate products (2–15 nm). Taking into account that the thermal conductivity of graphene-NMP NFs did not change with increasing graphene concentration, it seems that the degradation of NMP and the resulting organic contamination can affect the interaction between the additive nanomaterial (i.e., graphene) and the solvent, as well as the thermal performance of the NF. This causes a detrimental impact on the thermal conductivity of the nanofluid, in a manner that is still unknown.

## 4. Conclusions

In this work, a correlation between the modification of the vibrational spectra of the fluids, and the increase of their thermal conductivity and viscosity in the presence of graphene is presented, where the base fluids were DMAc, DMF, and NMP. Our results suggest that the presence of graphene produces important changes at the macroscopic level in DMF and DMAc-based fluids. This is reflected in the displacement of some Raman bands when the concentration of graphene increases. The blue-shift of several Raman bands in DMF and DMAc reveals a hardening of the bonds associated with those vibrational modes (intramolecular interactions), which requires higher energy to make them vibrate. In a way, it can be explained as if graphene has the ability to modify, as an external force, the solvent–solvent interaction, and fluid molecules, in turn, influence the intramolecular bonds of the neighbor molecules. This interpretation suggests that there is a strong modification in the intermolecular interaction between fluid molecules in the presence of graphene flakes, which in turn seems to be directly connected with the enhancement of the thermal conductivity of the fluid. In the case of NMP-graphene-based nanofluid no significant modification in the thermal conductivity was found. Similarly, we did not find an important modification in their Raman spectra either. The structure of the NMP molecule and the peculiar arrangement of the bare fluid (dimerization and inner order), can explain why the presence of graphene does not affect NMP in vibrational terms. NMP is a cyclic amide and has a strong tendency to form dimers in the liquid state, therefore, the modification of the intramolecular forces in the cycle would require great efforts. We can deduce from Raman spectroscopy and thermal conductivity results, that those concentrations of graphene are insufficient to impact on the fluid’s thermal properties. We tested a higher concentration of graphene in NMP (C = 0.5 mg/mL), but the thermal conductivity was not enhanced, and the Raman spectrum showed photoluminescence, which masks the Raman peaks. Photoluminescence can be attributed to the degradation of NMP during sonication, which is a crucial step in the preparation of stable dispersions. The sonochemical degradation of NMP, together with the formation of the small organic contaminating particulates, affects the interaction between the graphene flakes, which could also contribute to a detrimental impact on the thermal conductivity.

## Figures and Tables

**Table 1:** Thermal conductivity and viscosity of graphene based nanofluids.

Concentration		Samples					
		DMAc *		DMF *		NMP (This Work)	
mg/mL	wt%	*k* (W m^−1^ K^−1^)	Viscosity (mPa·s)	*K* (W m^−1^ K^−1^)	Viscosity (mPa·s)	*k* (W m^−1^ K^−1^)	Viscosity (mPa·s)
0.00	0	0.175	1.19	0.183	0.94	0.235	2.07
0.05	0.005	-	-	-	-	0.234	2.19
0.10	0.01	0.180	1.17	0.194	0.99	0.236	2.21
0.25	0.03	0.196	1.18	0.203	1.01	-	-
0.50	0.05	0.206	1.26	0.228	1.08	0.213	2.92
1.13	0.12	-	-	-	1.26	-	-
1.50	0.18	0.259	1.68	-	-	-	-

* Taken from Rodríguez-Laguna et al. [27].

## Supplementary Materials

The following are available online at https://www.mdpi.com/2079-4991/9/5/804/s1, Table S1: Summary of the Raman measurements taken on different days over a period of time greater than one year, Figure S1: Raman spectra of graphene DMAc-NFs recorded on different dates as a function of graphene concentration: (a) 0.1 mg/mL, (b) 0.2 mg/mL and (c) 0.5 mg/mL, Figure S2: Raman spectra of NMP-NFs recorded on different dates as a function of graphene concentration: (a) 0.05 mg/mL and (b) 0.1 mg/mL, Figure S3: Raman spectra of DMF-based nanofluids recorded on different dates as a function of graphene concentration: (a) 0.10 mg/mL, (b) 0.25 mg/mL and (b) 0.50 mg/mL.
