# Supplementary material for: Modification of the Raman Spectra in Graphene-Based Nanofluids and Its Correlation with Thermal Properties
Source: Nanomaterials (Basel). 2019 May 26;9(5):804. doi: 10.3390/nano9050804 (PMC6566612; doi:10.3390/nano9050804)
Supplement: Supplementary file 1 [file nanomaterials-09-00804-s001.pdf]

Modification of the Raman Spectra in Graphene-Based Nanofluids and Its Correlation with Thermal Properties

María del Rocío Rodríguez-Laguna [1,2,*], Pedro Gómez-Romero [1,*], Clivia M. Sotomayor Torres [1,3] and Emigdio Chavez-Angel [1,*]

[1] Catalan Institute of Nanoscience and Nanotechnology (ICN2), CSIC and The Barcelona Institute of Science and Technology (BIST), Campus UAB, Bellaterra, 08193 Barcelona, Spain; clivia.sotomayor@icn2.cat (C.M.S.T)
[2] Department of Chemistry, Universitat Autònoma de Barcelona, Campus UAB, Bellaterra, 08193 Barcelona, Spain
[3] ICREA- Institució Catalana de Recerca i Estudis Avançats, Pg. Lluís Companys 23, 08010 Barcelona, Spain.
* Correspondence: rodriguez3laguna@gmail.com (M.R.R.L.); pedro.gomez@icn2.cat (P.G.R.); emigdio.chavez@icn2.cat (E.C.A.); Tel.: +34 93 7373616 (M.R.R.L.); +34 93 7373608 (P.G.R.); +34 93 7371617 (E.C.A.)

Raman spectra of nanofluids as a function of time

In order to confirm the reproducibility of the measurements, Raman spectra of the three types of graphene nanofluids (DMAc, DMF and NMP- NFs) were recorded as a function of time for different concentrations of graphene. **Table S1** shows a summary of the set of measurements.

**Sample name**	**Date (day No.)**		
Graphene-DMAc NFs	2017/11/30 (day 1)	2017/12/22 (day 22)	2019/05/07 (day 524)
Graphene-NMP NFs	2018/02/05 (day 1)	2019/03/15 (day 404)	2019/05/08 (day 458)
Graphene-DMF NFs	2017/09/28 (day 1)	-	2019/05/08 (day 588)

Table S1 Summary of the Raman measurements taken on different days over a period of time greater than one year.

Figure S1, Figure S2 and Figure S3 show the Raman spectra of DMAc-, NMP- and DMF-based nanofluids for different graphene concentrations measured on different days, respectively. As can be seen, the Raman spectra do not change significantly as a function of time. We did not observe any modification and/or displacement of the Raman bands of the samples. Taking into account that the Raman spectra of nanofluids recorded after one year are practically identical to those performed on day 1, we can assure that the quality of the dispersion is maintained after more than one year. These results indicate the good quality and long-term stability of the samples.

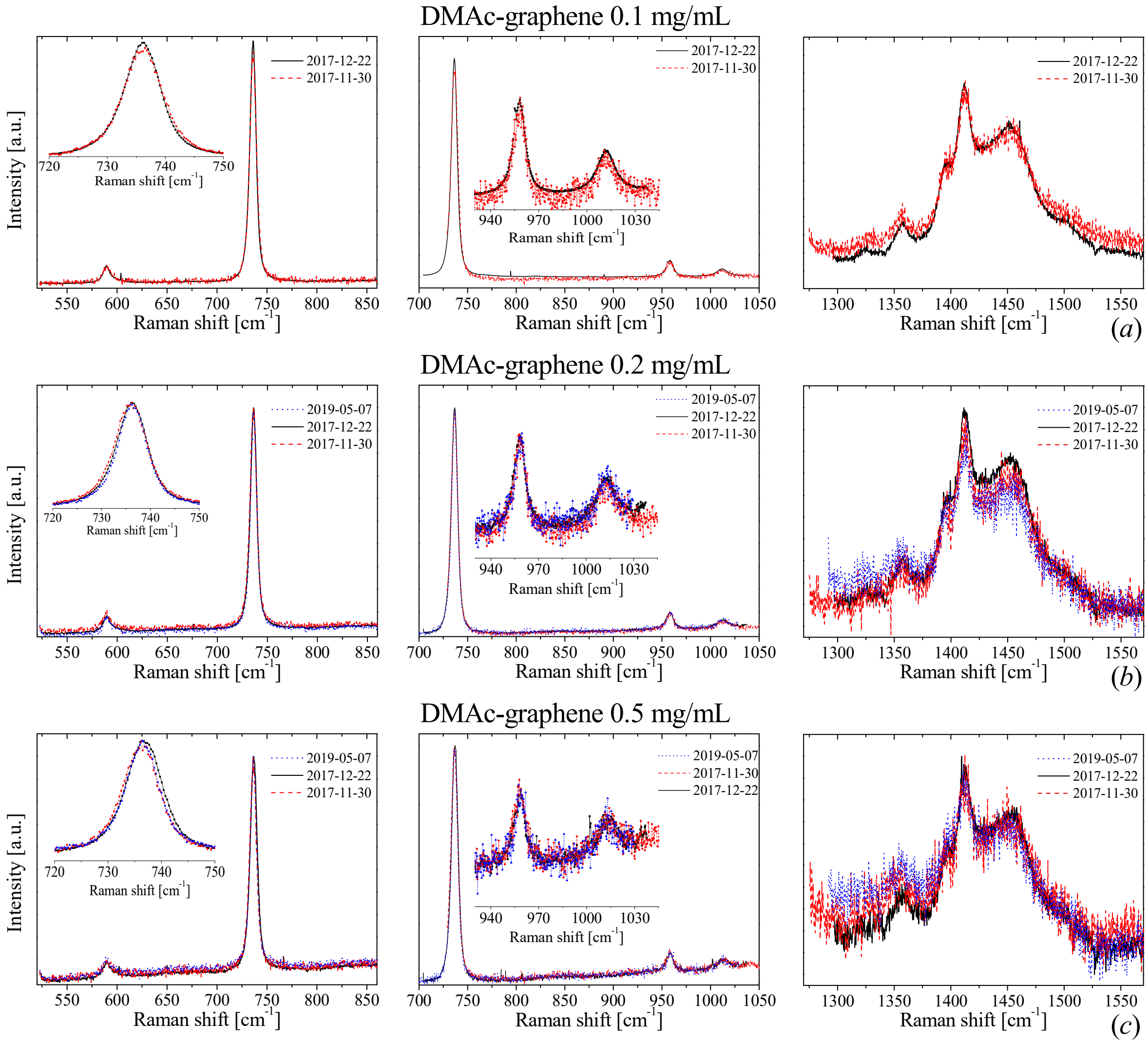

Figure S1 Raman spectra of graphene DMAc-NFs recorded on different dates as a function of graphene concentration: (a) 0.1 mg/mL, (b) 0.2 mg/mL and (c) 0.5 mg/mL.

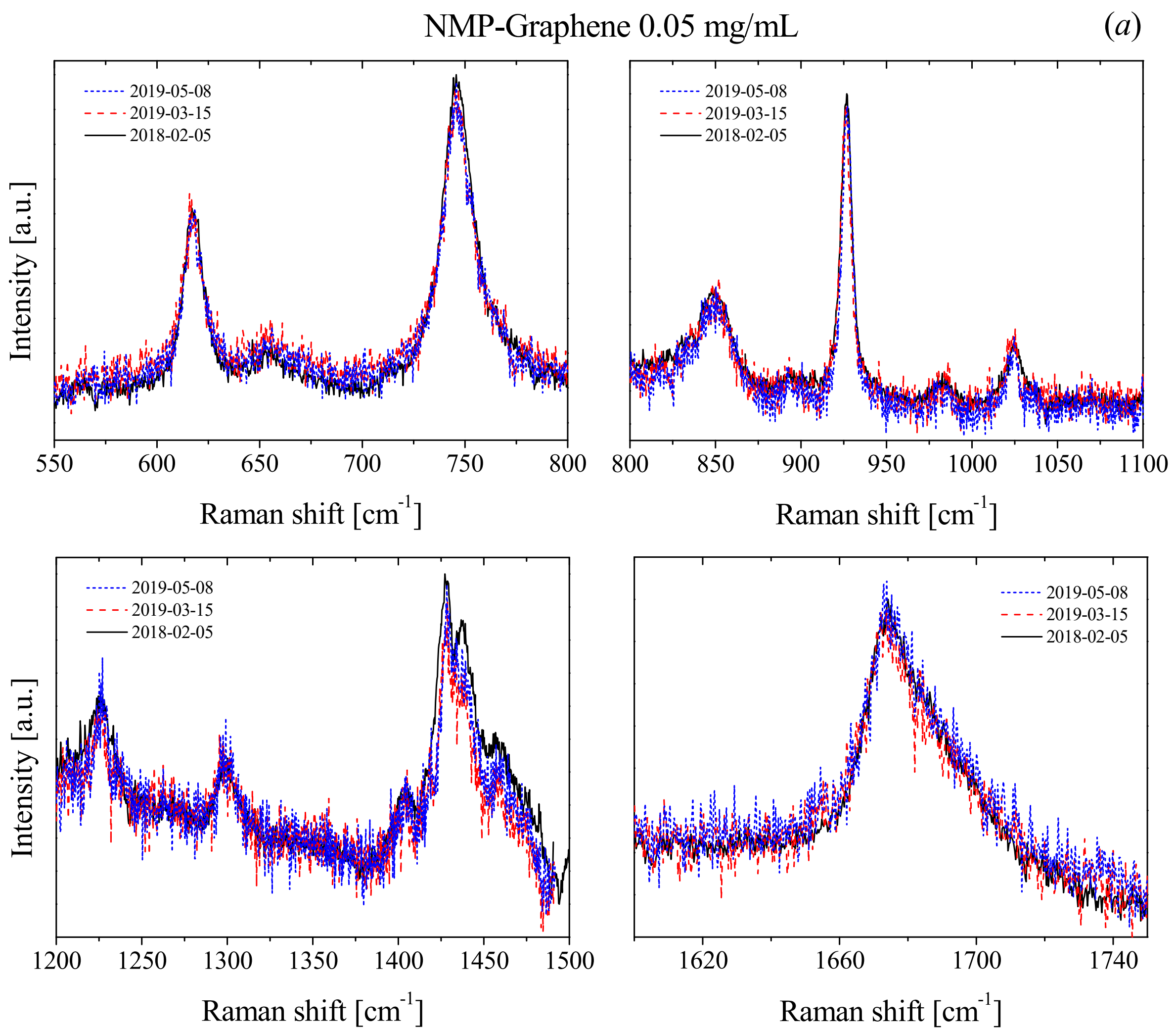
NMP-Graphene 0.05 mg/mL
(a)
2019-05-08
2019-03-15
2018-02-05
Intensity [a.u.]
Raman shift [cm-1]
550
600
650
700
750
800
850
900
950
1000
1050
1100
1200
1250
1300
1350
1400
1450
1500
1620
1660
1700
1740

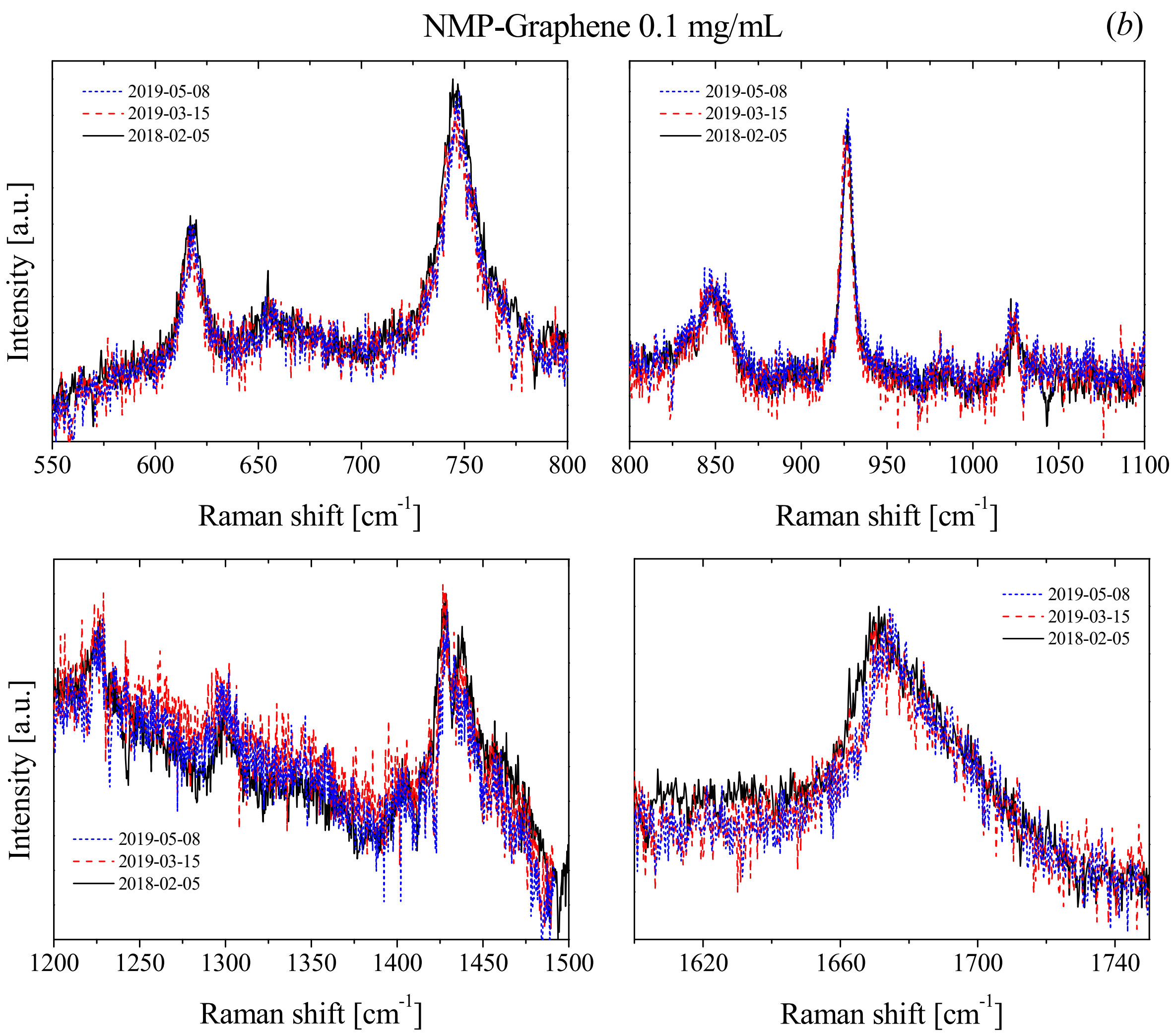

Figure S2 Raman spectra of NMP-NFs recorded on different dates as a function of graphene concentration: (a) 0.05 mg/mL and (b) 0.1 mg/mL.

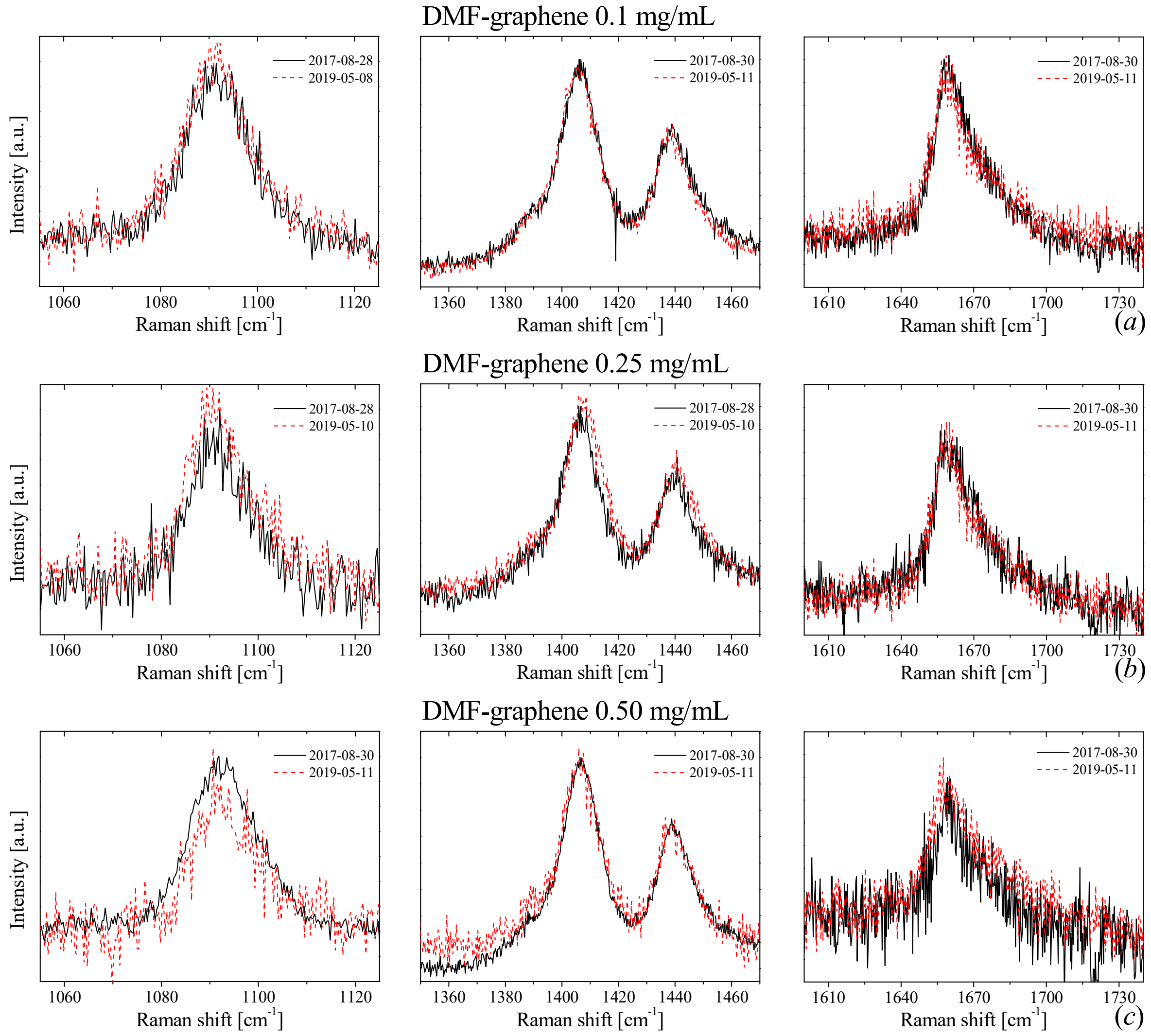

Figure S3 Raman spectra of DMF-based nanofluids recorded on different dates as a function of graphene concentration: (a) 0.10 mg/mL, (b) 0.25 mg/mL and (b) 0.50 mg/mL.
